# Characterization and molecular epidemiology of *Staphylococcus aureus* strains resistant to beta-lactams isolated from the milk of cows diagnosed with subclinical mastitis

**DOI:** 10.14202/vetworld.2019.1931-1939

**Published:** 2019-12-11

**Authors:** Geziella Áurea Aparecida Damasceno Souza, Anna Christina de Almeida, Mauro Aparecido de Sousa Xavier, Lívia Mara Vitorino da Silva, Cintya Neves Sousa, Demerson Arruda Sanglard, Alessandra Rejane Ericsson de Oliveira Xavier

**Affiliations:** 1 Federal University of Minas Gerais, Institute of Agrarian Sciences, Center for Research in Agrarian Sciences, Laboratory of Animal Health, Montes Claros, Minas Gerais, Brazil; 2 State University of Montes Claros, Center of Biological and Health Sciences, Microbiology Laboratory, Montes Claros, Minas Gerais, Brazil; 3 Federal University of Minas Gerais, Institute of Agrarian Sciences, Center for Research in Agrarian Sciences, Laboratory of Biotechnology, Montes Claros, Minas Gerais, Brazil

**Keywords:** beta-lactams, genetic diversity, infection, resistance genes, *Staphylococcus aureus*

## Abstract

**Background and Aim:**

The term ESKAPE, recognized by the WHO, is an acronym, which refers to the pathogens *Enterococcus faecium*, *Staphylococcus aureus*, *Klebsiella pneumoniae*, *Acinetobacter baumannii*, *Pseudomonas aeruginosa*, and *Enterobacter* spp., which is extremely virulent and multidrug-resistant. Although the term is used to designate nosocomial pathogens, in a milking environment, strains of Methicillin-resistant *S. aureus* have been isolated from cattle diagnosed with clinical and subclinical mastitis. Resistant strains may be involved in the transfer of genes conferring resistance to beta-lactam antimicrobials among the species of microorganisms related to mastitis etiology. This study aimed to trace the phenotypic and genotypic profiles of susceptibility to beta-lactams in *S. aureus* isolated from milk of cattle diagnosed with subclinical mastitis obtained from different rural properties located in the North of Minas Gerais State, Brazil.

**Materials and Methods:**

Sixteen microorganisms previously identified as *S. aureus* isolated from milk of cattle diagnosed with subclinical mastitis were submitted to matrix-assisted laser desorption/ionization-time-of-flight (MALDI-TOF), mass spectrometry, and polymerase chain reaction (PCR) analysis for microbial species confirmation. The *S. aureus* beta-lactams antimicrobial phenotypic resistance profile was investigated by disk diffusion method. PCR methods were also performed to investigate the *S. aureus* genotypic beta-lactams resistance profile. For this purpose, *bla*_Z_, *mec* A, *mec*_ALGA251_, *bla*_Oxa23_, and *bla*_KPC_ genes were screened among *S. aureus* isolates. The genetic diversity of *S. aureus* by fingerprint random amplified polymorphic DNA (RAPD)-PCR was also performed in this study.

**Results:**

All isolates showed phenotypic resistance to at least three beta-lactams, among which was meropenem. None of the isolates tested positive for the genes *mec*_ALGA251_, *bla*_Oxa23_, and *bla*_KPC_; however, the presence of the genes *bla*_Z_ and *mec*A was detected among the isolates. The fingerprint analysis divided isolates into two distinct groups and 15 different subgroups. Despite the presence of clonality among the isolates, the PCR-RAPD analysis unveiled a heterogeneous profile with genetic diversity among the *S. aureus* isolates.

**Conclusion:**

In this study, we identified beta-lactams resistant *S. aureus* strains isolated from the milk of cows diagnosed with subclinical mastitis. The *S. aureus* beta-lactams resistance was investigated using a phenotypic and genotypic approach. We believe that molecular epidemiology, improved knowledge, and genetic basis of resistance to beta-lactams might assist in asserting guidelines for better management practices of dealing with subclinical mastitis and mapping of origin of resistant pathogens in the studied Brazilian area.

## Introduction

*Staphylococcus aureus* is universally recognized as the causative agent of mammary glands infections in bovine herds [1-5]. Subclinical mastitis (SM) is the most prevalent infectious breast disease in dairy cows [6]. Although SM is characterized by the absence of clinical signs and several detrimental effects on milk production is noticed, its milk is marketed [2,6]. Programs for mastitis prevention and treatment include the use of antimicrobials, among which are the beta-lactams [5,7]. However, the indiscriminate use of antibiotics, which can lead to multidrug resistance, and increased risk of the presence of antimicrobial residues in milk, has made mastitis a disease of significant importance to public health [8,9]. *S*. *aureus* harbors genes that impart resistance to antimicrobial agents, which leads to complications in the treatment of their infections as well as increases the cost of treatments [3,7].

The resistance of *Staphylococcus* spp. to beta-lactams, specifically to methicillin in *S. aureus*, is quite advanced, and it is a widespread public health problem worldwide [10-13]. Two mechanisms have been described for the resistance to beta-lactams in the genus *Staphylococcus*. One of them is the production of the beta-lactamase enzyme encoded by the gene *bla*Z [11,14-17]. Another mechanism involves the synthesis of penicillin-binding protein 2A (PBP2A) with a low affinity for binding to penicillin coded by the gene *mecA* and its counterparts, among which *mec* ^*A*LGA251^ [10-12,16,18]. The presence of genes that encode the expression of carbapenem-type beta-lactamases has been described in *Acinetobacter* spp. [19,20] and *Klebsiella pneumoniae* [21,22]. These organisms are also potential etiologic agents of bovine mastitis [23-26], as well as disseminators of resistance genes to other pathogens [27]. In *Acinetobacter baumannii*, resistance to carbapenem agents can be attributed to the presence of the gene *bla*_Oxa23_ [19,20] and to the gene *bla*_KPC_ in *K. pneumoniae* [21,22]. The presence of the gene *bla*_Oxa23_ has already been reported in pathogens other than *A. baumannii*, confirming the transferability of this gene to other species of microorganisms [28,29]. Strains of *Escherichia coli*, *Enterobacter* spp., *Salmonella* spp., *Acinetobacter* spp., and *Pseudomonas* spp. carrying plasmids containing the gene *bla*_KPC_ have also been reported in countries such as the United States, Israel, China, Brazil, and the European continent [30-32].

Molecular epidemiological studies have contributed to the mapping of sources, routes of transmission, and prognosis for many pathogens that cause bovine mastitis, as well as to the understanding of the adaptation mechanisms of the host and the causes of the disease [7,33,34]. Quick and simple techniques based on identifying fingerprints of repetitive elements have been described for studies of genetic diversity and epidemiological analysis of the genus *Staphylococcus* [7,34-38]. Considering this background, this study aimed to trace the phenotypic and genotypic profiles of susceptibility to beta-lactams in *S. aureus* isolated from bovine milk of herd with subclinical mastitis from different rural properties located in the North of Minas Gerais, Brazil. The analysis of the genetic diversity of isolates of *S. aureus* by fingerprint random amplified polymorphic DNA (RAPD) - polymerase chain reaction (PCR) was also included in this study.

## Materials and Methods

### Ethical approval

This work was performed within the ethical standards approved by the Ethics and Animal Experimentation of Federal University of Minas Gerais (UFMG) under protocols number 145/2013 and 90/2018.

### Bacterial isolates and profile of sensitivity to beta-lactam antimicrobials

Sixteen samples of *S. aureus* maintained in the cell bank of the Animal Health laboratory of the Institute of Agrarian Sciences at the Federal University of Minas Gerais were selected. The samples of *S*. *aureus* were isolated from the milk of cows diagnosed with subclinical mastitis originating from seven rural properties in the North of Minas Gerais, Brazil: Janaúba (FL[15°48’13’’S], and NP[43°19’3’’W]), Porteirinha (MU[15°44’38’’S]), Icaraí de Minas (VA[16°11’46’’S]), Matias Cardoso (FH[14°51’20’’S]), São João da Lagoa (SL[16°46’42’’S]), and Bocaiuva (TR[17°6’55’’S]). The identification of isolates at the species level was performed by detection of the gene *femA*, a species-specific marker for *S. aureus* as described by Xavier *et al*. [7].

The susceptibility to beta-lactam antibiotics was determined by the disk diffusion method according to the Clinical and Laboratory Standards Institute guideline [39] using the following antimicrobials (Laborclin): Amoxicillin – 10 μg (AMO), cefoxitin – 30 μg (CFO), oxacillin – 1 μg (OXA), ampicillin – 10 μg (AMP), and meropenem – 10 μg (MER). A standard strain of *S. aureus* ATCC 25923 was used as positive control.

### Confirmation of the identification of *S. aureus* to species level by MALDI-TOF MS

The cryopreserved isolates genotyped as *S. aureus* were reactivated by sowing in Brain Heart Infusion (BHI) agar medium (BHI Broth – Kasvi) and incubating at 37°C for 24 h. The selected colonies were individually added to a steel plate. Then, to this plate, 1 μl of formic acid (70%) and 1 μl of MALDI-TOF MS matrix were added, consisting of a saturated solution of a-cyano-4-hydroxycinnamic acid (Bruker Daltonics, Bremen, Germany), and it was left to air dry. The spectra were obtained using the mass spectrometer MicroFlex LT (Bruker Daltonics) with a nitrogen laser of 60 Hz, in which up to 240 laser photos are triggered in spiral movements to collect 40 steps of shot for each point of tension. In addition, the parameters for detection of mass range were defined to allow the identification from 1960 to 20,137 m/z, where the source of ions 1 v was 19.99 kv, the ion source voltage of 2 kv was 18.24, and the lens voltage was 6.0 kv for data acquisition (manufacturer’s specifications). Previous the identification tests, the equipment was calibrated using a standard bacterial test (*E. coli* DH5 alpha; Bruker Daltonics). The criteria of the identification score in real-time used were those recommended by the manufacturer: ≥2000 score indicates identification at the species level, score ≥1700 and <2000 indicate identification at the level of genus, and <1700 score indicates absence of reliable identification. The comparisons between the identifications of the strain by MALDI-TOF MS and other techniques were performed using the R software version 3.0.1 (R Core Team, 2013) with the concordance rates determined by the Kappa coefficient.

### DNA extraction

The cryopreserved isolates of *S. aureus* were reactivated by sowing in the BHI medium (Prodimol Biotecnologia) and incubating at 37°C for 24 h. Growth bacterial cultures were subjected to DNA extraction, as described by Gu *et al*. [40]. The integrity and quantity of extracted DNA were verified by electrophoresis in 1.0% agarose gel. This material was used in the PCR and RAPD-PCR reactions performed in this study. All primers used in this study were synthesized by Genome Biotechnologies, Brazil.

### PCR analysis for the detection of 16S rDNA gene

The presence of the universal 16S bacterial rDNA gene was verified by PCR with the primers DG74 5’AGGAGGTGATCCAACCGCA3’ and RW01 5’AACTGGAGGAAGGTGGGGAT3’ generating an amplicon of approximately 370 base pairs [9]. The reactions were carried out in a mix containing 2× Go Taq Green Master Mix^®^ (Promega, Corporation, USA), MgCl_2_ (2.5 mm), 10 µM of each primer, and 50 ng of DNA in a final total reaction volume of 50 µl. The PCR reaction was performed in a Thermal Cycler Veriti (Applied Biosystems, California, USA). The thermal conditions of PCR amplification were performed according to Xavier *et al*. [9]. The amplicons were visualized in 1.5% agarose gel stained with ethidium bromide and photodocumented. As a positive control, a standard strain of *S. aureus* ATCC 43300 was used, and sterile water was used as a negative control.

### PCR analysis for the detection of genes *bla*_KPC_, *bla*_OXA23_, blaZ, *mec*A, and *mec*_ALGA251_ related to resistance to the tested beta-lactams

The presence of the genes *bla*_KPC_, *bla*_OXA23_, *bla*Z, *mec*Am and *mec*_ALGA251_ was verified by PCR. The sequences of the gene targets to be detected and the sizes of the expected fragments amplified by the reaction are indicated in Table-1 [10,14,18,19,21]. The reactions were carried out in a mix containing 2× Go Taq Green Master Mix^®^ (Promega, Corporation, USA), MgCl_2_ (2.5 mm), 10 µM of each primer, and 50 ng of DNA in the final total reaction volume of 50 µl. The PCR reaction was performed in a Thermal Cycler Veriti (Applied Biosystems, California, USA). The PCR thermal conditions were those described by the authors added in Table-1. The amplicons were visualized in 1.5% agarose gel stained with ethidium bromide and photodocumented. As a positive control for the detection of the *bla*Z, *mec*A, and *mec*_ALGA251_, a standard strain of *S. aureus* ATCC 43300 was used. For detection of the *bla*_KPC_ and *bla*_OXA23_ genes, DNA of nosocomial isolates of *K. pneumoniae* and *A. baumannii* (genotyped by Ezequiel Dias Foundation from Minas Gerais, Brazil) kindly donated by the microbiology laboratory of State University of Montes Claros were used, respectively. In all the reactions, sterile water was used as a negative control.

**Table-1:** List of primers, sequence, gene target, the amplicon expected size, and references for PCR analysis for the detection of genes related to beta-lactams resistance.

Primer	Sequence (5’ … 3’)	Target gene	Amplicon	References
KPC- 1a KPC- 1b	TGTCACTGTATCGCCGTC CTCAGTGCTCTACAGAAAACC	*bla*_KPC_	~879 pb	[21]
OXA 23-F OXA 23-R	ATGTGTCATAGTATTCGTCG TCACAACAACTAAAAGCACTG	bla_OXA23_	~1057 pb	[19]
BLAZ-1 BLAZ-2	TTAAAGTCTTACCGAAAGCAG TAAGAGATTTGCCTATGCTT	*bla*_Z_	~377 pb	[14]
mecAL1 mecAL2	TCACCAGGTTCAAC[Y]CAAAA CCTGAATC[W]GCTAATAATATTTC	*mec*_ALGA251_	~356 pb	[10]
mecA1 mecA2	AGTTCTGCAGTACCGGATTTGC AAATCGATGGTAAAGGTTGGC	*mec*A	~533 pb	[18]

PCR=Polymerase Chain Reaction

### RAPD-PCR and statistical analysis

The genetic profile characterization and identification of the clonal relationship among isolates of *S. aureus* were performed using the RAPD-PCR technique. For the RAPD-PCR reaction, the oligonucleotide S232 was used, and PCR conditions were as described in the literature for the analysis of polymorphism within and among species of *S. aureus* [7,35]. The reactions were carried out in a mix containing 1× Buffer of Taq of Kappa PCR Kit, MgCl_2_ (2.5 mm), deoxynucleotides (1 µM), Taq Polymerase Invitrogen, (0.5 U), 1 µM of each primer, and 5 µl (50 ng/µl) of bacterial DNA in a final total reaction volume of 25 µl. The amplicons were visualized in 1.8% agarose gel stained with ethidium bromide and photodocumented. The analysis of the amplification of DNA profiles obtained by RAPD-PCR was performed by visual inspection of two observers and transformed into binary data in an array, according to the presence (1) or (0) absence of bands. To assess the genetic relationship among the isolates, the matrix was subjected to a multivariate “Cluster Analysis” by the method of full thread (complete linkage) for the calculation of the Euclidean distance and generation of a dendrogram in the statistical program Minitab v.16. (Minitab, USA). As a positive control, a standard strain of *S. aureus* ATCC 43300 was used and sterile water was used as a negative control.

### Sequencing of PCR products of genes blaZ and mec_ALGA251_

The PCR products corresponding to the optimization of PCR reactions with the genes *bla*Z and *mec*_ALGA251_ were sequenced by the Sanger method (Ludwig Biotech, RS, Brazil – ACTGene Análises Moleculares) using the primers described in Table-1. To obtain the amplified sequence by PCR, the sequencing tapes were aligned with the Clustal Omega tool (European Bioinformatics Institute – https://www.ebi.ac.uk/Tools/msa/clustalo/), and differences were visualized on an electropherogram (Chromas v.2.6.5 – www.technelysium.com.au) and corrected. After obtaining the amplified sequence, it was used for the BLAST completion (https://blast.ncbi.nlm.nih.gov/Blast.cgi). After the blast, the result obtained was aligned with the sequence resulting from the BLAST using the Clustal Omega tool (European Bioinformatics Institute – https://www.ebi.ac.uk/Tools/msa/clustalo/).

## Results and Discussion

### Phenotypic profile of resistance to beta-lactam antimicrobials

All the isolates proved to be resistant to OXA and CFO (16/16). The resistance index to AMO among isolates was 87.5% (14/16), and ten isolates (62.5%) showed resistance to AMP. The resistance to MER was found in 56.25% (9/16) of the isolates (Table-2).

**Table-2:** Phenotypic and genotypic profile of resistance to antimicrobials and analysis of genetic diversity of 16 *Staphylococcus aureus* isolated from milk of cows with subclinical mastitis collected in different rural properties in the North of Minas Gerais.

Number and code of the Isolate		Date of isolation	Place of isolation	MALDI-TOF phenotypic identification	Genotypic identification		Phenotypic profile of resistance to anti microbials	Genotypic profile of resistance to antimicrobials					Genetic Profile PCR RAPD	
		
					Gene16S rDNA	Gene *fem*A		*Gene bla*Kpc	*Gene bla*OXA_23_	Gene *bla*Z	Gene *mec*A	*Gene mecA_LGA251_*	Group	Genotype
1	FH 1	12/01/2014	MC	*S. aureus*	+	+	MER, CFO, OXA	-	-	NT	-	-	I	IA
2	MU 1	01/07/2016	P	*S. aureus*	+	+	MER, CFO, OXA, AMP, AMO	-	-	+	+	-	II	IIA
3	MU 2	01/07/2016	P	*S. aureus*	+	+	CFO, OXA, AMO	NT	NT	-	-	-	I	IG
4	SL 1	01/12/2016	SJL	*S. aureus*	+	+	CFO, OXA, AMO	NT	NT	-	-	-	II	IIB
5	SL 2	01/12/2016	SJL	*S. aureus*	+	+	CFO, OXA, AMO, AMP	NT	NT	-	-	-	II	IID
6	SL 3	01/12/2016	SJL	*S. aureus*	+	+	CFO, OXA, AMO, AMP	NT	NT	-	-	-	II	IIC
7	VA 1	01/28/2016	IM	*S. aureus*	+	+	MER, CFO, OXA, AMP, AMO	-	-	+	+	-	I	IB
8	VA 2	01/28/2016	IM	*S. aureus*	+	+	MER, CFO, OXA,	-	-	NT	-	-	I	IE
9	FL1	02/25/2016	J	*S. aureus*	+	+	MER, CFO, OXA, AMP, AMO	-	-	+	+	-	I	IC
10	FL 2	02/25/2016	J	*S. aureus*	+	+	MER, CFO, OXA, AMP, AMO	-	-	+	+	-	I	IC
11	FL 3	02/25/2016	J	*S. aureus*	+	+	MER, CFO, OXA, AMP, AMO	-	-	+	-	-	II	IIE
12	FL 4	02/25/2016	J	*S. aureus*	+	+	CFO, OXA, AMO, AMP, AMO	NT	NT	-	-	-	I	ID
13	FL 5	02/25/2016	J	*S. aureus*	+	+	CFO, OXA, AMO	NT	NT	-	-	-	II	IIF
14	NP 1	2/26/2016	J	*S. aureus*	+	+	MER, CFO, OXA, AMP, AMO	-	-	+	+	-	I	IF
15	NP 4	2/26/2016	J	*S. aureus*	+	+	CFO, OXA, AMO	NT	NT	-	-	-	I	IH
16	TR 3	04/19/2016	B	*S. aureus*	+	+	MER, CFO, OXA, AMP, AMO	-	-	-	-	-	II	IIG
17	PC	-	-	*S. aureus*	+	+	CFO, OXA, AMO, AMP	NT	NT	+	+	+	I	IF

AMP=Ampicillin, AMO=Amoxicillin, CFO=Cefoxitin, OXA=Oxacillin, MER=Meropenem, NT=Not tested. MC Montes claros, P=Porteirinha, SJL=São João da Lagoa, IM=Icaraí de Minas,; J (Janaúba); B (Bocaiuva). PC=Positive control (*S. aureus* ATCC 4330). More details on materials and methods. *S. aureus*=*Staphylococcus aureus,* PCR=Polymerase chain reaction, RAPD=Random amplified polymorphic DNA

The profile of simultaneous resistance to MER, CFO, OXA, AMP, and AMO was found in 43.75% of the isolates (7/16). Four isolates (25%) presented with the resistance profile of CFO, OXA, and AMO. The resistance profiles CFO, OXA, AMO, AMP and MER, CFO, and OXA were found in 62.5% (10/16) and 56.25% (9/16) of the isolates. *S. aureus* resistant to OXA, AMO, AMP, and CFO was isolated from animals with mastitis [7,41-45]. Rossi *et al*. [44], over a period of 12 months, isolated 116 strains of *S. aureus* from the milk of cows diagnosed with subclinical mastitis on farms in the region of Piracicaba, in the state of São Paulo, Brazil. Among these isolates, there was a low rate of resistance to the antimicrobials OXA (7.8%) and CFO (4.3%). However, strains of *Staphylococcus* spp. isolated from milk of cows with mastitis on farms in Rio Grande do Sul, Brazil, presented with profiles of resistance to AMO (50%) and AMP (43.3%), similar to those found in our study [42]. The high rates of resistance to OXA, AMO, AMP, and CFO, and multidrug resistance identified here reveals the persistence and widespread dissemination of these beta-lactam resistant bacteria in cows with subclinical mastitis in the studied region. Freitas *et al*. [42] concluded that the multidrug resistance of *S. aureus* to antimicrobials is due to their external use, empirical, or improper use of these drugs for the treatment and/or prophylaxis of diseases in the bovines’ mammary glands.

Carbapenems are beta-lactam antibiotics which are effective against both Gram-negative and Gram-positive bacteria [46]. However, carbapenems are not the first choice drug in clinical mastitis treatment [46]. Gram-negative microorganisms, such as *E. coli*, *K. pneumoniae*, and *A. baumannii*, are described in the literature as pathogens involved in the mastitis etiology [23-26]. The possibility of antimicrobial resistance genes transferring between the same species and different species in a milking environment sheltering cows with subclinical mastitis led us to investigate the susceptibility of *S. aureus* isolates to MER. The results of the disk diffusion test revealed that more than 50% of the *S. aureus* tested showed phenotypic resistance to MER (Table-2). The resistance mechanisms of Gram-negative germs to carbapenem antibiotics are known [47]; however, there are reports of this resistance being present in Gram-positive bacteria, among which is *S. aureus*. To the best of our knowledge, this is the first time in literature that the phenotypic resistance to carbapenems has been reported in strains of *S. aureus* isolated from the milk of bovines diagnosed with subclinical mastitis.

### Identification of *S. aureus* and genotypic analysis of resistance to beta-lactam antimicrobials

The evaluation of the quality of the DNA extracted and the confirmation of the bacterial isolates were performed by PCR of the universal 16S rDNA region. All the isolates amplified a fragment of approximately 370 bp corresponding to the 16S rDNA bacterial universal gene. The confirmation of the species *S. aureus* at a genomic level was previously asserted by the presence of the *fem*A gene marker in the samples, according to Xavier *et al*. [9]. All isolates and standard strains tested positive for the gene *fem*A. In addition to the gene *fem*A, the *nuc* gene and *16S rDNA gene* have also been reported as species-specific markers for *S. aureus* and are recognized as robust tests for the identification of the species of this microorganism [5,41,48].

The MALDI-TOF MS analysis of isolates confirmed the results of the genomic analysis, where all the isolates exhibited a proteomic profile of *S*. *aureus* (Table-2). This method has been successfully used for the identification of *S. aureus* isolated from samples of bovine milk [5,48].

**Figure-1:**
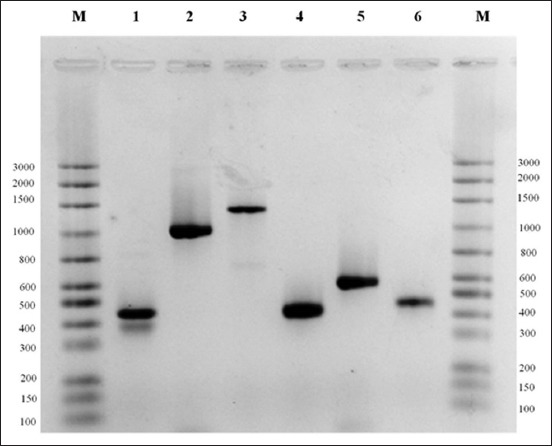
Polymerase chain reaction (PCR) optimization with positive controls for universal bacteria *16S rDNA*, *bla_OXA23,_*bla*_KPC,_*bla*_Z_, mecA, and mec*_ALG250_ genes *_._* M: Molecular weight marker *Mid-Range* (Cellco Biotecnologia), Line 1: PCR *Staphylococcus aureus* strain ATCC 43300 for detection of universal bacteria 16S rDNA gene (~370pb). Line 2: PCR nosocomial *Klebsiella pneumoniae* isolate for detection of *bla_KPC_* gene (~876pb). Line 3: PCR *Acinetobacter baumannii* isolate for detection of *bla_OXA23_* gene (~1057pb) Line 4: PCR *S. aureus* strain ATCC 43300 for detection of *bla_Z_* gene (~377pb). Line 5: PCR *S*. *aureus* strain ATCC 43300 for detection of *mecA* gene (533pb *)*. Line 6: PCR *S. aureus* strain ATCC 43300 for detection of gene *mec*_ALGA251_ (~356pb). Electrophoresis on 1.5% agarose gel.

The PCR standardization for the detection of resistance to antimicrobials was performed in standard strains and nosocomial isolates containing the genes studied here. Figure-1 shows the result of the optimization of the genes *bla_KPC_, *bla*_OXA23_, blaZ, mecA*, and *mec_ALGA251_* related to beta-lactam resistance.

The PCR sequencing results of *S. aureus* ATCC 43300 standard strain corresponding to the gene *bla*Z revealed that it presented 100% similarity to a partial sequence of the gene *bla*Z of *S. aureus* stored in GenBank (access number: DQ16050). The PCR product of the strain pattern of *S. aureus* corresponding to *mecA* and *mec*_ALGA251_ genes was also sequenced and showed similarity of 91% and 96% to a partial sequence of the gene *mecA* that encodes for a PBP2A of strain of Methicillin-resistant *S. aureus* (MRSA) stored in GenBank access numbers MH798869.1 and MH798864.1, respectively. The results of the sequencing of PCR products corresponding to the genes *mecA*, *mec*_ALGA251_, and *bla*Z analyzed here have confirmed that these genes have approximate sizes of amplified fragments to those cited by the authors of these primers (Table-1 and Figure-1). After the standardization of PCR with standard strains, the *S. aureus* isolates were submitted to specific PCR for the screening of the genes *bla*_KPC_, *bla*_OXA23_, *bla*Z, *mec*A, and *mec*_ALGA251_ related to beta-lactam resistance. All the isolates that showed phenotypic resistance to the antimicrobial MER were subjected to PCR for the screening of the genes *bla_KPC_, *bla*_OXA23_* related to resistance to carbapenems. None of the nine tested isolates amplified the genes *bla*_KPC_ and/or *bla*_OXA23_ in the PCR reactions (Table-2). Until the present moment, no report of *S. aureus* carrying the *bla*_KPC_ and/or *bla*_OXA23_ genes has been described in the literature corroborating with the results obtained here. However, the screening of other genes related to the presence of resistance to carbapenems in these samples, among which are those described by Codjoe and Donkor [47], could be a strategy to be used to elucidate the *in vitro* resistance of these isolates to MER.

Isolates resistant to AMO and AMP were subjected to a PCR screening for the gene *bla*Z. The presence of an amplicon corresponding to a region of the *bla*Z gene was detected in 41.8% isolates (6/14) and in the tested standard strain of *S. aureus* ATCC 43300 (Table-2). Although the phenotypic resistance to AMP has been found in 14 isolates, only six tested positive for the presence of the gene *bla*Z. The same isolates that tested positive for the gene *bla*Z presented simultaneous resistance phenotypic profiles to AMO (6/10) (Table-2). Strains of *S. aureus* isolated from bovines with mastitis were already described in literature as bearers of the genes *bla*Z, *blaI*, and *blaR* [14,17,48]. Qu *et al*. [34] detected the presence of the gene *bla*Z in 95% of the *S. aureus* strains isolated from the milk of cows with mastitis in different regions of China.

The resistance of *S. aureus* to penicillin and its analogs is explained by the production of the enzyme β-lactamase, encoded by a regulatory cluster composed of the genes *blaZ*, *blaI*, and *blaR* [17]. The results of phenotypic resistance to AMO and AMP whose *bla*Z gene was not detected may be explained by the emergence of a mutation in this gene in *S. aureus* samples tested here. The search for the gene regions *blaI* and *blaR* in these samples could determine the genetic origin of resistance to these beta-lactams.

Isolates resistant to OXA and CFO were subjected to PCR for the screening of genes *mec*A and its counterpart *mec*_ALGA251_. Although the phenotypic resistance to OXA and CFO has been found in 100% of the isolates (16/16), only 31.25% (5/16) tested positive for the presence of gene *mecA* related to methicillin/OXA/CFO resistance. None of the isolates tested positive for the *mec*_ALGA251_ gene which is homologous to the gene *mecA*. Dias *et al*. [18] also obtained divergence between the phenotypic and genotypic results associated with *S. aureus* resistance to OXA. Although 55.5% of *S. aureus* isolates have proved to be phenotypically resistant to OXA, only 11% carried the *mecA* gene. The authors justify the genotypic resistance to methicillin/OXA found in *S. aureus* isolates to the presence of homologous genes to *mecA*, among which is *mec*C. Rossi *et al*. [44] reported the presence of gene *mecA* in four of the nine *S. aureus* phenotypically resistant to OXA isolated from milk of a herd with subclinical mastitis. None of these isolates tested positive for the gene *mec*C. The *mec*C gene, also known as gene *mec*_ALGA251_, was identified in *S. aureus* isolates from both humans and animals [10,12,44,49]. This gene has 70% of homology to the *mec*A gene [10]. According to Rolo *et al*. [12], the β-lactams resistance mediated by *mecA* can be explained through four distinct mechanisms: Accumulation of substitutions in a specific domain of β-lactamase; diversification of the gene promoter; and acquisition of a new cassette Staphylococcal Cassette Chromosome *mec* and adaptation to genetic background.

### Analysis of the genetic diversity of *S. aureus*

**Figure-2:**
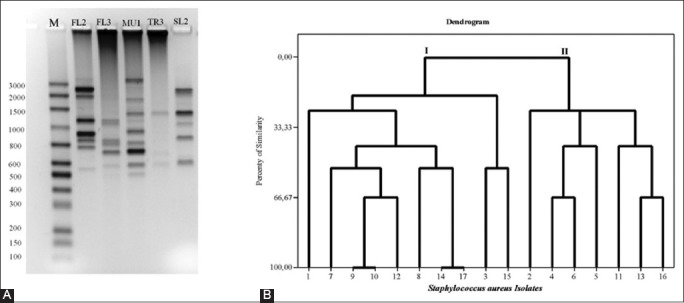
Random amplified polymorphic DNA (RAPD)-polymerase chain reaction (PCR) and dendrogram of genetic relationship of the *Staphylococcus* isolated from milk of bovines diagnosed with subclinical mastitis. Panel A: Electrophoresis on 1.8% agarose gel containing RAPD-PCR amplicons of five isolates of *Staphylococcus aureus*. M: Molecular weight marker *Mid-Range* (Cellco Biotecnologia), Line 1 to 5: RAPD-PCR of *S. aureus* FL2, FL3, MU1, TR3, and SL2 isolates. Panel B: Dendrogram of genetic relationship of the *Staphylococcus aureus* (n=16) isolated from milk of bovines diagnosed with subclinical mastitis. Dendrogram of genetic relationship of the *Staphylococcus aureus* (n=16) isolated from milk of bovines diagnosed with subclinical mastitis. The 16 isolates were divided into two groups (I and II) according to the degree of similarity based on the Euclidean distance calculation. The RAPD-PCR profile of standard strain of *S. aureus* ATCC 43300 was used for the dendrogram generation (corresponding to 17).

In this study, the analysis of genetic diversity of 16 isolates resistant to beta-lactams using the RAPD technique produced 19 different fragments (490 bp, 500 bp, 510 bp, 600 bp, 610 bp, 700 bp, 750 bp, 790 bp, 800 bp, 810 bp, 900 bp, 1000 bp, 1100 bp, 1400 bp, 1500 bp, 1900 bp, 2000 bp, 2500 bp, and 3000 bp). For the bands sharing analysis, the bands in a range between 450 and 3000 base pairs were considered (Figure-2A).

The binary matrix made by visual observation of the presence or absence of these bands after being subjected to cluster analysis for the Euclidean distance calculation generated a dendrogram of genetic relationships among isolates of *S*. *aureus* (Figure-2B). These were grouped into two different groups of which 56.25% (9/16) showed a profile of Group I and 43.75% (7/16) of Group II. Still, according to the Euclidean distance calculation, it was possible to determine 15 different genotypes (IA to IH and IIA to IIG), which revealed heterogeneity of genotypes of *S. aureus* not only among isolates from different rural properties but also in those isolated within the same herd (Figures-2A and B, Table-2). The genetic diversity of *S. aureus* obtained from cows’ milk from others had similar results to this work on analyzing through the PCR-RAPD technique [7,36,38]. However, clonality was verified (100% of similarity) between the isolates 9 (FL1) and 10 (FL2) obtained from the same rural property in the municipality of Janaúba. The comparison of the genetic relationships among these isolates showed convergence between the phenotypic profiles of sensitivity to antibiotics (MER, CFO, OXA, AMP, and AMO) and the characterization of the genetic profile by polymorphism analysis based on PCR-RAPD-(IC) (Table-2). A degree of similarity (100%) was also found between the isolate 14 (NP1) and the standard strain of *S. aureus* ATCC 43300 used for validation of the PCR-RAPD method. Even though both of them presented the same phenotypic antibiotic resistance profile, the only difference presented between *S. aureus* ATCC 43300 and isolate 14 was related to detection of amplicon to the gene *mec*_ALGA251_ (Table-2).

Epidemiological studies have contributed to the clarification of the sources and routes of pathogen transmission related to mastitis [7,34]. Pathogenic strains can be transferred from one animal to another by different sources, including by the animal itself. The transmission path includes contact between animals, beds of creation, milking equipment, and the milkers’ hands [7,33].

## Conclusion

It is possible to conclude from this work that strains of *S. aureus* isolated from the milk of herds diagnosed with subclinical mastitis showed phenotypic resistance to the tested beta-lactams. In addition, strains carrying genes related to beta-lactam resistance, among which *bla*Z and *mecA*, were reported. Hence, the *S. aureus* (MRSA) found here deserves special attention, once the milk in the region surveyed is marketed and used for human consumption *in natura* or even for the production of artisan cheese. The presence of MER resistant strains may indicate the misuse of antimicrobials in herds in the studied region. The PCR-RAPD analysis showed a heterogeneous profile among isolates in different rural properties. Studies with a greater number of *S. aureus* samples collected from the milk of cows diagnosed with subclinical mastitis during longer periods will be necessary for the better elucidation of the molecular epidemiology in the studied region. In particular, the elucidation of the mechanisms of gene transfer conferring resistance to antimicrobial agents in herds, especially those treated but remain with subclinical mastitis that is not diagnosed, since they are a potential reservoir of microorganisms that cause infection. The gene flow mapping of antimicrobial resistance and genes related to toxin production in milking environments can provide useful information for the management of clinical and subclinical mastitis in animal production.

## Authors’ Contributions

GAADS, ACA, MASX, DAS and AREOX designed the study. LMVD and CNS collected the samples. GAADS, CNS, and ACA performed the experiments. GAADS, ACA, and AREOX performed data analysis. AREOX and GAADS wrote the manuscript. All authors edited, read, and approved the final manuscript.
